# Preparation and Investigation of Silver Nanoparticle–Antibody Bioconjugates for Electrochemical Immunoassay of Tick-Borne Encephalitis

**DOI:** 10.3390/s19092103

**Published:** 2019-05-07

**Authors:** Yekaterina Khristunova, Elena Korotkova, Bohumil Kratochvil, Jiri Barek, Elena Dorozhko, Vlastimil Vyskocil, Evgenii Plotnikov, Olesya Voronova, Vladimir Sidelnikov

**Affiliations:** 1 Department of Chemical Engineering, National Research Tomsk Polytechnic University, Lenin Avenue 30, 634050 Tomsk, Russia; eph2@tpu.ru (Y.K.); eikor@mail.ru (E.K.); bohumil.kratochvil@vscht.cz (B.K.); elena-dorozhko@yandex.ru (E.D.); plotnikov.e@mail.ru (E.P.); oaa@tpu.ru (O.V.); vladimir.svibla.sidelnikov@gmail.com (V.S.); 2 Faculty of Science, Department of Analytical Chemistry, UNESCO Laboratory of Environmental Electrochemistry, Charles University, Albertov 6, 12843 Prague 2, Czech Republic; vlastimil.vyskocil@natur.cuni.cz; 3 Department of Solid State Chemistry, University of Chemistry and Technology, Prague, Technicka 5, 16628 Prague 6, Czech Republic

**Keywords:** Bioconjugates, silver nanoparticles, antibodies, tick-borne encephalitis virus, cathodic linear sweep voltammetry (CLSV), gold–carbon composite electrode (GCCE), electrochemical immunosensor

## Abstract

A new simple electrochemical immunosensor approach for the determination of antibodies to tick-borne encephalitis virus (TBEV) in immunological products was developed and tested. The assay is performed by detecting the silver reduction signal in the bioconjugates with antibodies (*Ab@AgNP*). Here, signal is read by cathodic linear sweep voltammetry (CLSV) through the detection of silver chloride reduction on a gold–carbon composite electrode (GCCE). Covalent immobilization of the antigen on the electrode surface was performed after thiolation and glutarization of the GCCE. Specific attention has been paid to the selection of conditions for stabilizing both the silver nanoparticles and their *Ab@AgNP*. A simple flocculation test with NaCl was used to select the concentration of antibodies, and the additional stabilizer bovine serum albumin (BSA) was used for *Ab@AgNP* preparation. The antibodies to TBEV were quantified in the range from 50 IU·mL^−1^ to 1600 IU·mL^−1^, with a detection limit of 50 IU·mL^−1^. The coefficient of determination (*r*^2^) is 0.989. The electrochemical immunosensor was successfully applied to check the quality of immunological products containing IgG antibodies to TBEV. The present work paves the path for a novel method for monitoring TBEV in biological fluids.

## 1. Introduction

Tick-borne encephalitis virus (TBEV) is one of the endemic flaviviruses in Russia, which can cause serious infections in humans that may result in encephalitis/meningoencephalitis. Among the flaviviruses, TBEV has one of the highest impacts as a human pathogen. Each year, up to 10,000 human cases are reported in Russia [1]. TBEV is a widespread zoonotic virus infection characterized by fever and brain grey matter damage (encephalitis) and/or damage to meninges (meningitis and meningoencephalitis). The disease may cause persistent neurological and psychiatric damage with lethal outcome [2]. After TBEV infection, specific cellular and humoral responses are developed, and as a result, the production of antibodies to this pathogen starts. The most frequently used protective treatment for tick-borne encephalitis (TBE) within 96 h after a tick bite is the usage of the immunoglobulins against TBEV (passive immunization) [3,4]. The amount of antibody product is normalized in relation to the phase of the disease and the age of the person. Therefore, it is important to monitor immunological products containing antibodies to TBEV that are used as post-exposure prophylaxis after a tick bite. For the successful diagnosis of TBE, detection of antibodies to this pathogen in human blood can be preferably used. Nowadays, enzyme-linked immunosorbent assays (ELISAs) are the most frequently used methods of choice for this purpose (while nucleic acid tests are most frequently applied to detect TBE viral RNA in ticks) [5]. Each ELISA kit includes a conjugate based on antibodies and marker enzymes. The procedure for conjugates preparation is multistage and complex, which leads to a significant cost of diagnostic kits. The significant loss of enzyme and immunoglobulins activity is possible (from 30 to 50%) in the process of their covalent crosslinking. Furthermore, marker enzymes are stored only at low temperatures or in preservative solutions, which leads to the need for periodic evaluation of their activity [6].

Electrochemical immunosensors utilizing inexpensive and more stable colloids of metal nanoparticles (NPs) for the labelling of immunoreagents can overcome the previously described disadvantages of ELISA and can thus be used as a suitable alternative. Recent publications confirm that there is an increased interest in the development of electrochemical immunological methods for TBE detection [7,8,9,10,11,12,13]. Table 1 summarizes types of electrodes, modifiers, and electrochemical labels applied for TBE determination.

From Table 1, it can be seen that in the majority of publications based on the determination of TBEV antigens, impedance electrochemical sensors with a sandwich format without labels were used [9,10,11,12,13]. In these works, the resistance at the electrode in the presence of redox probes before and after the formation of the antigen-antibody complex on the electrode surface is measured [9,10,11,12,13]. Most of them utilize a more complex and time- and labour-demanding technique for electrode modification. For voltammetric determination of antibodies to TBEV, authors [7,8] proposed the use of colloids of gold or silver conjugated with protein A. However, information about dispersion, production, and stabilization of colloids and their bioconjugates is missing in these publications. Moreover, the figures of merits of the proposed voltammetric methods are not comparable with the results of the reference method (ELISA).

In this paper, the bioconjugate of Ag nanoparticles (NPs) and antibodies to TBEV (*Ab@AgNP*) have been prepared and reported for the first time. Here, Ag is a direct signalling marker that is monitored using CLSV that relies on the AgCl reduction. This electrochemical strategy for the detection of antibodies to TBEV has been employed also for the first time. Specific attention has been paid to the selection of conditions for the stabilization of both Ag NPs and their *Ab@AgNP* bioconjugates.

The electrochemical immunosensor utilizing *Ab@AgNP* bioconjugates for the determination of antibodies to TBEV has been developed. The comparative detection of IgG antibodies to TBEV in immunological products was carried out using the developed electrochemical immunosensor and an ELISA method for the first time. The electrochemical immunosensor presented here represents a proof of concept for the oncoming development of such progressive diagnostic tools applicable for biological fluids.

## 2. Materials and Methods

### 2.1. The Synthesis and Characterization of Ag NPs

Stable colloid silver was obtained by the method of Mulfinger and Solomon [14]. 5 mL of 1.0 mmol·L^−1^ AgNO_3_ were slowly pipetted (1 drop/s) into 15 mL of 2.0 mmol·L^−1^ NaBH_4_ at 4 °C under vigorous stirring with a magnetic stirrer. All solutions were dissolved in nanopure water (18 MΩ·cm). After Ag NP formation has been completed, the solution colour has changed to light yellow. To remove contaminations, Ag NPs were dialyzed through a dialyzing membrane (pore size of 1 kDa). The Ag NP solution was stable for 14 days during storage in chemical glassware (dark glass) at 4 °C.

After the dialysis has been completed, Ag NP absorption spectra were recorded on the Cary 2000 spectrophotometer (Agilent, Waldbronn, Germany) in 1.0 cm quartz cuvettes in the UV/Vis spectral region between 300 and 500 nm. The zeta potential of Ag NPs was measured on the Zetasizer Nano ZS (Malvern Panalytical, Westborough, MA, USA). Transmission electron microscopy (TEM) observations of the Ag NPs were performed with the GEOL GEM-2100F transmission electron microscope (provided by TPU Nano-Center, Tomsk, Russia). Calculations of the average size of the Ag NPs were done by scientific TEM image analysis with the Fiji program and Origin Pro 8.0 (OriginLab, Northampton, MA, USA). At least ten representative images were taken for each sample. A particle size distribution was obtained by counting at least 100 particles for each sample.

### 2.2. Stabilizing Ag NPs with BSA

Bovine serum albumin (BSA, Sigma-Aldrich, St. Louis, MO, USA, cat. No. A2153) solutions of different concentrations were added into a set of 2 mL Eppendorf tubes containing 1.9 mL of post-dialysis colloid Ag NP solutions (10 µg·mL^−1^). In the reaction volume, BSA concentration was as follows (µg·mL^−1^): 2.0, 4.0, 8.0, 16.0, 32.0. One tube without the BSA addition was used as a reference. The solutions were incubated at 37 °C for 1 h [15].

### 2.3. Flocculation Test

Flocculation test was used to check the stability of Ag NPs modified with BSA. 200 µL of 10% (*w*/*w*) NaCl were added into each tube. The tubes were shaken for 5 min; the fluid was visually monitored for colour changes and clouding. Afterwards, the tubes were centrifuged for 5 min at 16,000 rpm at 4 °C, and the fluid was again visually monitored for colour changes, clouding, or flocculates.

Moreover, after the centrifugation, the spectra of the obtained solutions were recorded in the UV/Vis spectral region between 300 and 500 nm. The size and morphology of the Ag NPs stabilized by BSA were determined using TEM.

### 2.4. Preparation of Ab@AgNP

The Ag NPs solution (5 mL) obtained and then purified by dialysis was centrifuged at 16,000 rpm for 10 min at 4 °C. The supernatant was removed, and the residue was resuspended in 2 mL of 0.1 mol·L^−1^ phosphate buffer (pH 7.4) containing antibodies to TBEV (10 µg·mL^−1^) (ELISA kit, Vector-Best, Novosibirsk, Russia). After an hour-long incubation and gentle stirring at 37 °C, the sample was centrifuged again with subsequent resuspending of the residue in 2 mL of the 0.1 mol·L^−1^ phosphate buffer solution with 16 µg·mL^−1^ of BSA. *Ab@AgNP* bioconjugate solutions were stored at 4 °C. High-molecular proteins are mostly used as stabilizers to prevent the aggregation of Ag NPs and protect them from the coagulating action of electrolytes [16]. The morphology of *Ab@AgNP* bioconjugates was determined using TEM.

Another type of bioconjugates with non-tick-borne monoclonal diagnostic antibodies (*Ab_1_@AgNP*, monoclonal Anti-β-Actin antibody produced in mouse (Sigma-Aldrich, St. Louis, MO, USA, cat. No. A5441) was obtained to conduct control experiments and to exclude the non-specific binding to the TBEV antigen. Those bioconjugates were prepared by the same methodology as the *Ab@AgNP* bioconjugates.

### 2.5. Production of an Electrochemical Immunosensor Based on Ab@AgNP Bioconjugates

Applying a gold–carbon composite electrode (GCCE) was a prerequisite to making of an electrochemical immunosensor. To fabricate the substrate electrode, gold nanoparticles were deposited electrochemically on the surface of a solid carbon composite electrode (CCE, disc-shaped, 3.9 mm in diameter, manufactured by Tomanalyt LLC, Tomsk, Russia) from a HAuCl_4_ solution (1000 mg·L^−1^). The conditions of the gold nanoparticle deposition on the CCE were as follows: scan rate of 5 mV·s^−1^, potential sweep ranging from −0.05 to −0.1 V [17,18]. Then, the electrode has been pre-treated by cyclic voltammetry in 0.5 mol·L^−1^ H_2_SO_4_ in the potential range between −1.5 and +1.5 V until reproducible cyclic voltammograms were obtained [19].

**Figure 1:**
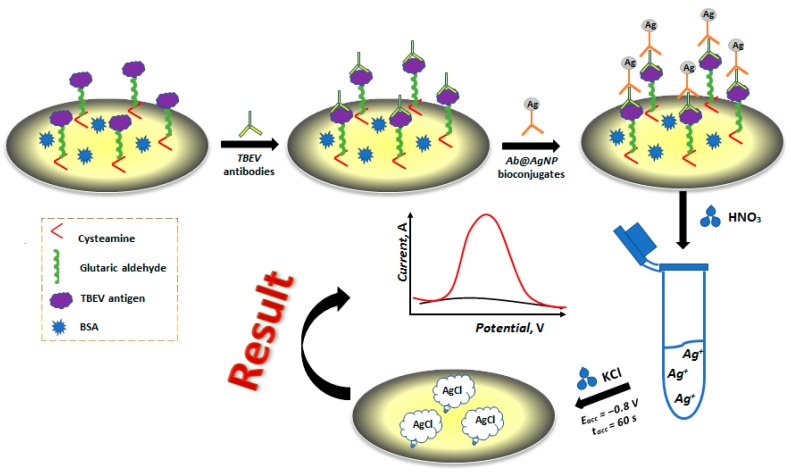
Scheme of the electrochemical immunosensor preparation and principle of antibody detection.

Figure 1 depicts the preparation of the electrochemical immunosensor and detection principle of antibodies. During the preliminary stage, the thiolation of the GCCE surface was performed by dipping it into 2 mL of a cysteamine solution (0.05 mol·L^−1^) for 45 min at room temperature. After rinsing the electrode with deionized water, the electrode was placed into a glutaric aldehyde solution (2.5%, *w*/*w*) for 45 min at room temperature. Glutaric aldehyde was used for covalent protein binding with the antigen through cysteamine amino groups and antigen protein [19]. Afterwards, the electrode has been rinsed with the phosphate buffer (pH 7.4) three times and the antigen with a volume of 20 µL was immobilized on the electrode surface (ELISA kit, Vector-Best, Novosibirsk, Russia). The electrode incubation time was 1 h at 24–26 °C. Then, the electrode has been rinsed with deionized water and immersed into a BSA solution (1%, *w*/*w*) for 30 min in order to block the sites of non-specific binding of the sensor with other non-specific proteins [20]. The obtained electrode with the immobilized antigen on the surface has been stored at 4 °C for 6 months without any change in performance.

After immobilizing the TBEV antigen on the GCCE surface, the electrode was placed into an anti-TBE virus ELISA "Vienna" IgG (Vector-Best, Novosibirsk, Russia) antibodies solution for 1 h at 37 °C. TBEV antibodies concentrations were as follows (IU·mL^−1^): 50, 100, 400, 800, 1600. Afterwards, the electrode has been rinsed with the phosphate buffer (pH 7.4) three times and the electrode was put into the *Ab@AgNP* bioconjugate solution for 1 h at 37 °C. Further investigation was based on the measurement of electrochemical signal from Ag in *Ab@AgNP* bioconjugates by cathodic linear sweep voltammetry (CLSV).

The control experiments included the same steps of producing the electrochemical immunosensor, with except of antigen immobilization; whole surface was blocked by BSA. Furthermore, *Ab_1_@AgNP* bioconjugates with monoclonal Anti-β-Actin antibody, which are non-specific to the TBEV antigen, were tested, and no voltammetric signal indicating the AgCl reduction on the GCCE was obtained.

### 2.6. Electrochemical Detection of Ag in Ab@AgNP Bioconjugates

For the detection of *Ab@AgNP* bioconjugates, CLSV was used in a three-electrode cell. The GCCE was used as the working electrode, an Ag|AgCl (1 mol·L^−1^ KCl) with a salt bridge to prevent chloride ions from entering the cell was used as a reference electrode, and a platinum wire electrode was used as an auxiliary electrode. The TBEV antibodies detection process was based on the preliminary dissolution of Ag in 1 mL of 1 mol·L^−1^ HNO_3_ over 15 min. Then, Ag was detected on the bare GCCE via the emergence of AgCl in the background electrolyte that contained Cl^−^ ions. Voltammetric measurements were carried out on the TALab analyzer (Tomanalyt LLC, Tomsk, Russia). The voltammetric parameters for the AgCl detection via its reduction signal were as follows: supporting electrolyte of 0.15 mol·L^−1^ HNO_3_ and 0.01 mol·L^−1^ KCl, scan rate of 100 mV·s^−1^, potential range from +0.6 to −0.15 V, accumulation potential of −0.8 V, accumulation time of 60 s.

### 2.7. Application of Electrochemical Immunosensor to the Analysis of Immunological Products

The performance of the newly developed immunosensor was tested for the determination of immunoglobulins against TBEV in two immunological products: human immunoglobulins against TBEV (FSUC SIC "Microgen", Moscow, Russia) with concentrations not less than 80 and 160 IU·mL^−1^. The electrochemical measurements were carried out as described in Section 2.6.

The ELISA (Vector-Best, Novosibirsk, Russia) was selected as a comparative method, where the detection is based on the indirect assay. The amount of the bound conjugate (horseradish peroxidase-labelled antibodies to TBEV) with human immunoglobulin against TBEV is determined by colour reaction using a peroxidase substrate-hydrogen peroxide and 3,3′,5,5′-tetramethylbenzidine. The intensity of staining is proportional to the concentration of antibodies to TBEV in the immunological product.

## 3. Results

### 3.1. Characterization of Ab@AgNP Bioconjugates

**Figure 2:**
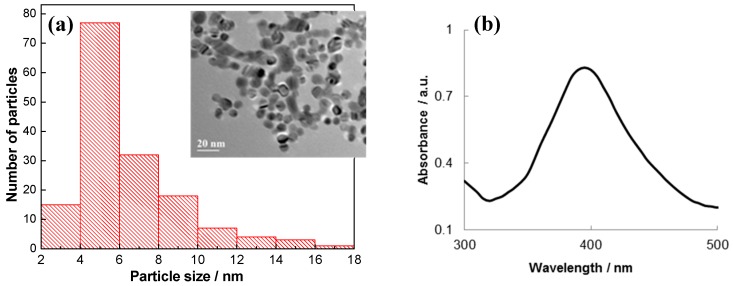
TEM-derived Ag nanoparticle (NP) size distribution and TEM image of Ag NPs (inset) (**a**); UV/Vis absorption spectra of clear yellow colloidal Ag (Ag NPs), optical path length of 1.0 cm, blank—deionized water **(b).**

At first, spherically shaped Ag NPs (5.3 ± 1.2 nm in size) were synthesized, purified by dialysis, and characterized by TEM and UV/Vis spectrophotometry (Figure 2). In the UV/Vis absorption spectra (Figure 2b), the maximum absorption of Ag NPs is in the range of 395–400 nm, which is in accordance with the average Ag NP size of 5.3 ± 1.2 nm calculated from the TEM observations (Figure 2a) [14]. The zeta potential of the Ag NPs was found to be −42 mV. This large negative zeta potential value indicates repulsion among the Ag NPs and their dispersion stability.

Afterwards, stable and active *Ab@AgNP* bioconjugates applicable for electrochemical immunoassay [16] were prepared in several steps: (i) the empirical optimization of the minimum concentration of BSA used for additional Ag NP stabilization and for blocking excess Ag NPs to prevent non-specific binding was carried out; (ii) bioconjugates were produced by the Ag NP incubation with antibodies to TBEV in the reaction medium containing BSA; (iii) the removal of non-bound antibodies and BSA from the *Ab@AgNP* bioconjugate was carried out by centrifuging and resuspending. BSA was chosen because it is inert, inexpensive, and it can inhibit Ag NP aggregation/agglomeration for 2 months at room temperature.

**Figure 3:**
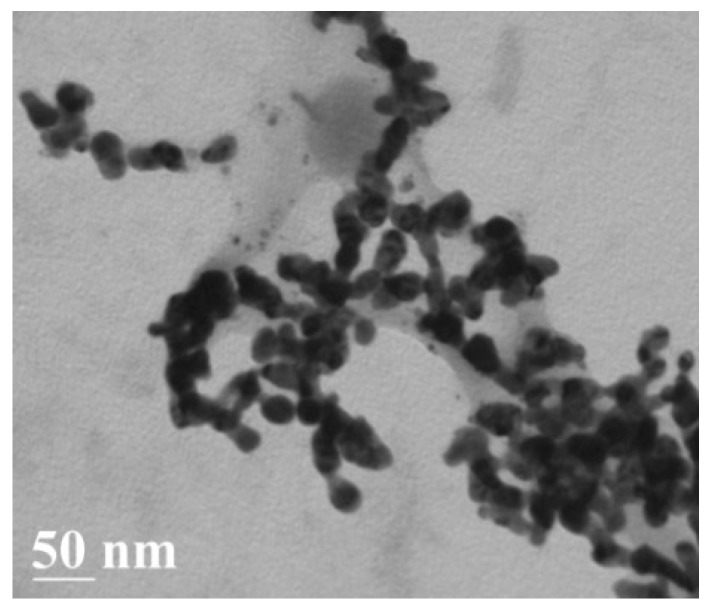
TEM image of *Ab@AgNP* bioconjugates.

Antibodies that are specific to the TBEV were taken from ELISA kit (Vector-Best, Novosibirsk, Russia). Figure 3 shows a TEM image of *Ab@AgNP* bioconjugates. The stability of *Ab@AgNP* bioconjugates was confirmed by an experiment one month later giving the same results.

### 3.2. Optimization of BSA Concentration for Stabilization of Ag NPs

**Figure 4:**
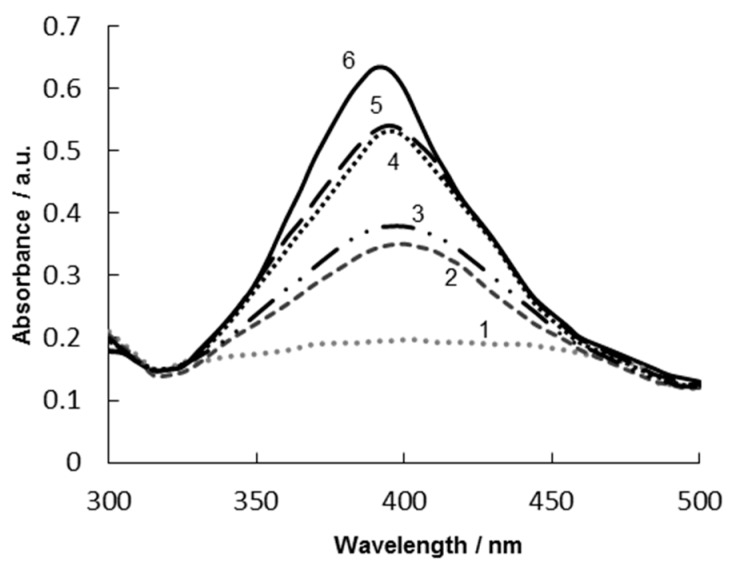
UV/Vis absorption spectra of Ag NPs **6** and after NaCl addition **1–5** and subsequent centrifugation in the presence of different concentrations of BSA: **1**—2 µg·mL^−1^, **2**—4 µg·mL^−1^, **3**—8 µg·mL^−1^, **4**—16 µg·mL^−1^, **5**—32 µg·mL^−1^; optical path length of 1.0 cm, blank—deionized water.

In order to empirically optimize the minimum BSA concentration for stabilizing Ag NPs, flocculation test was used. Different BSA concentrations were added into a series of tubes containing a Ag NP suspension, and the mixture was incubated as described above. Afterwards, 200 µL of 10% (*w*/*w*) NaCl (destabilizing agent) were added into each tube, and centrifuging was used as an extra destabilization factor, too. The samples, in which protein concentration was insufficient, exhibited signs of Ag NP aggregation. The colour of pure colloid silver solution was light yellow. If Ag NPs were aggregating, the solution colour changed to grey [14]. Figure 4 shows the Ag NP absorption spectra with different BSA concentrations after the flocculation test with centrifugation.

It can be seen that BSA addition shifts the maximum of the UV/Vis absorption spectra of Ag NP to longer wavelengths (400–405 nm) as compared to the absorption maximum of the Ag NPs without the BSA (Figure 4, curve 6). It confirms that BSA stabilizes the Ag NPs, probably via several amino acids present in BSA (e.g., histidine, cysteine, aspartic and glutamic acid), which promote its binding with metal salts. In particular, moieties of BSA with sulphur-, oxygen-, and nitrogen-bearing groups can stabilize the nanoparticles. Of these, thiol-bearing cysteine residues are likely to interact with Ag NPs more strongly via direct chemical bonding, thus providing steric stabilization [21].

The BSA concentration of 2 µg·mL^−1^ is not high enough to stabilize the Ag NPs with BSA in the presence of the destabilizing agent (NaCl) even without centrifugation. In Figure 4, curve 1, no absorption maximum corresponding to a complex of the Ag NPs with BSA can be observed. After the NaCl addition, the Ag NPs are instantly deposited and the solution becomes fully transparent. The BSA concentrations of 4 µg·mL^−1^ (Figure 4, curve 2) and 8 µg·mL^−1^ (Figure 4, curve 3) appear to be sufficient to protect the Ag NP colloid from aggregation, but the Ag NPs are still deposited after the sample centrifugation. The BSA concentrations of 16 µg·mL^−1^ and 32 µg·mL^−1^ are high enough to stabilize the Ag NP colloid even after centrifugation (Figure 4, curves 4–5). Thus, the agreement of the absorption spectra of the Ag NPs in the presence of BSA in the concentrations of 16 µg·mL^−1^ and 32 µg·mL^−1^, as well as the flocculation test with centrifugation, have both demonstrated that the BSA concentration of 16 µg·mL^−1^ is high enough to stabilize the Ag NPs for 45 days.

**Figure 5:**
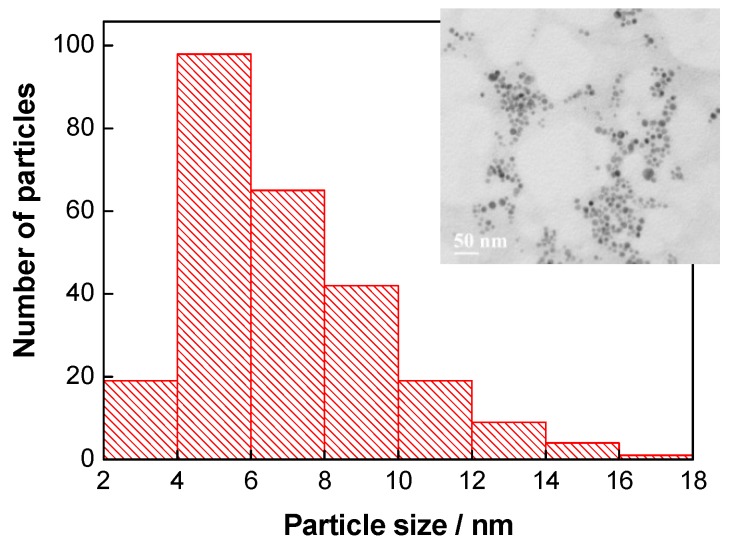
TEM-derived Ag NP size distribution in the presence of blocking reagent (BSA) (16 µg·mL^−1^) and TEM image of Ag NPs in the presence of BSA (16 µg·mL^−1^) (inset).

The obtained Ag NPs stabilized by BSA (16 µg·mL^−1^) were investigated by TEM as shown in Figure 5. Obviously, BSA is acting as a stabilizer of Ag NPs.

### 3.3. Electrochemical Determination of Antibodies to TBEV

The produced *Ab@AgNP* bioconjugates can be used in diagnostics as an analytical tool for the determination of antibodies to TBEV. To assess the quality of the produced *Ab@AgNP* bioconjugates, an electrochemical immunosensor was developed and CLS voltammograms of Ag in the *Ab@AgNP* bioconjugates as a direct signalling marker were recorded. The preparation and characterization of the electrochemical immunosensor were performed in several steps and is presented in Figure 1.

**Figure 6:**
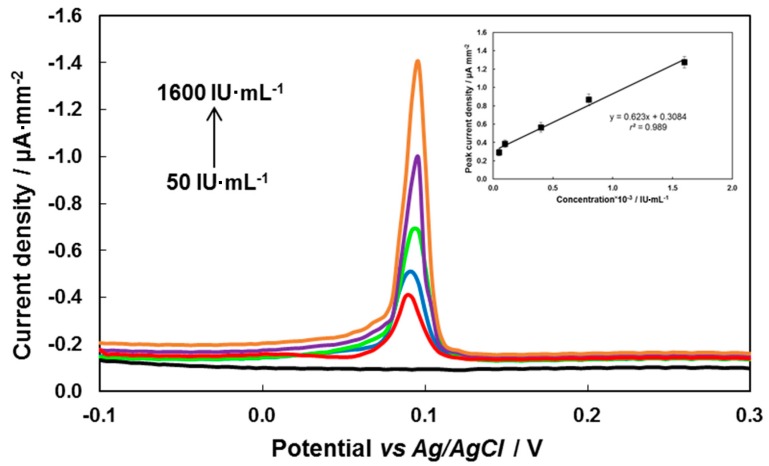
Cathodic linear sweep voltammograms of AgCl at the gold–carbon composite electrode (GCCE); black line—without the antigen immobilized on the immunosensor surface; coloured lines—antigen immobilized on the electrochemical immunosensor with the different concentration of antibodies (50, 100, 400, 800, 1600 IU·mL^−1^). Inset plot: corresponding calibration curve (error bars for *n* = 5, *P* = 0.95) in supporting electrolyte of 0.15 mol·L^−1^ HNO_3_ and 0.01 mol·L^−1^ KCl. Scan rate of 100 mV·s^−1^, *E*_acc_ = −0.8 V, *t*_acc_ = 60 s.

Figure 6 shows CLS voltammograms of the AgCl reduction on the GCCE when the antigen was present on the electrochemical immunosensor surface (Figure 6, coloured). Free silver ions were prepared by dissolving metallic silver in 1 mol·L^−1^ HNO_3_, and then AgCl (formed by precipitation of Ag^+^ with Cl^−^) was reduced at the potential of +0.1 V on the bare GCCE, thus generating the signal [22]. In the case of the comparative experiments without the immobilized antigen on the surface of the electrochemical immunosensor and with another type of bioconjugates (*Ab_1_@AgNP*), no electrochemical signal indicating the AgCl reduction on the GCCE was detected (Figure 6, black line).

The concentration range of this electrochemical immunosensor is from 50 IU·mL^−1^ to 1600 IU·mL^−1^ TBEV antibodies (i.e., the same as corresponding ELISA kit) with a detection limit of 50 IU·mL^−1^. The detection limit was calculated as LOD = 3*s*/*b* (where *s* is the standard deviation the signal of blank sample; *b* is the slope of the straight section of the calibration curve) [23].

The practical applicability of the newly developed immunosensor was verified by the determination of the concentration of immunoglobulins against TBEV in two immunological products to verify the reliability of *Ab@AgNP* bioconjugates. ELISA (Vector-Best, Novosibirsk, Russia) was used as a comparative method (Table 2).

The obtained results clearly demonstrate that the detected concentrations of immunoglobulins against TBEV using the developed electrochemical sensor correspond to the values declared by the manufacturer and they agree with the results of the traditionally used ELISA method.

## 4. Conclusions

This study has shown that the obtained silver-labelled *Ab@AgNP* bioconjugates can be used in voltammetric immunoassays to determine antibodies to TBEV. Colloidal silver, as a label, is less expensive and more stable than enzyme labels. The procedure for colloidal silver labelling is very simple and includes the selection of the concentration of antibodies and a blocking reagent (BSA). Flocculation test with NaCl allows choosing the optimal concentration of antibodies and BSA for obtaining *Ab@AgNP* bioconjugates. The selection of the antibody and BSA concentration should be carried out in relation to the specific experimental conditions, the research objectives associated with the method of preparation and the dispersion of colloidal silver, the pH of the medium, the presence of impurities, etc. CLSV has confirmed that the *Ab@AgNP* bioconjugates can be used as an analytical tool for the quantitation of antibodies to TBEV in the concentration range from 50 IU·mL^−1^ to 1600 IU·mL^−1^, with a detection limit of 50 IU·mL^−1^. The newly developed electrochemical immunosensor was successfully applied to check the quality of immunological products containing immunoglobulins against TBEV.

## Figures and Tables

**Table 1:** Survey of electrochemical methods for determination of tick-borne encephalitis (TBE).

Electrode	Modifier	Method/Label	Target	Linearity Range (mg·mL^−1^)	Limit of Detection (ng·mL^−1^)	Ref.
Thick-film graphite electrode	Glutaric aldehyde, Nafion, or nitrocellulose	Anodic stripping voltammetry/protein A with Au NPs	Antibodies	10^−7^ − 10^−2^	0.1	[7]
Screen-printed electrode	-	Linear sweep voltammetry/protein A with Ag NPs	Antibodies	10^−7^ − 10^−2^	0.5	[8]
Platinum electrode	Nano-Au/*o*-phenylenediamine polymer film with deposited Prussian blue	Amperometry/label-free	Antigen	^a^ 1.1∙10^−^^8^ – 1.9∙10^−^^6^	^a^ 6∙10^−^^9^	[9]
Gold disc electrode	l-cysteine + nano-Au and [Co(bpy)_3_]^3+^	Potentiometry/label-free	Antigen	^a^ 8.1∙10^−^^8^ − 3.0∙10^−^^6^	^a^ 3.5∙10^−^^8^	[10]
Platinum microelectrode	Polyaniline/multiwalled carbon nanotubes	Electrochemical impedance spectroscopy/label-free	Antigen	2.0∙10^−^^6^ − 2.5∙10^−^^4^	-	[11]
Screen-printed electrode	Carbon nanoparticles modified with chitosan	Electrochemical impedance spectroscopy/label-free	Antigen	1.0∙10^−^^6^ − 2.0·10^−^^5^	0.36	[12]
Screen-printed electrode	Silanized surface with protein A/glutaric aldehyde	Electrochemical impedance spectroscopy/label-free	Antigen	10^−^^3^ − 10^−^^2^	750	[13]

^a^ data expressed in PFU mL^−^^1^ (plaque-forming units per milliliter).

**Table 2:** Comparative results of measuring concentrations of immunoglobulins against TBEV in immunological products using existing ELISA and the developed electrochemical method (*n* = 5, *P* = 0.95).

Immunological Product	Declared by Producer (IU·mL^−1^)	Found by ELISA (IU·mL^−1^) (C1)	Found by Electrochemical Method (IU·mL^−1^) (C2)	C2/C1 (%)
Human immunoglobulin against TBEV (FSUC SIC “Microgen”, Russia)	Not less than 80	86 ± 4	87 ± 4	101
Human immunoglobulin against TBEV (FSUC SIC “Microgen”, Russia)	Not less than 160	172 ± 8	165 ± 4	96
